# MiR-141-3p promotes mitochondrial dysfunction in ovariectomy-induced sarcopenia via targeting Fkbp5 and Fibin

**DOI:** 10.18632/aging.202617

**Published:** 2021-02-03

**Authors:** Hyunjung Lee, Young In Kim, Farida S. Nirmala, Ji-Sun Kim, Hyo-Deok Seo, Tae Youl Ha, Young-Jin Jang, Chang Hwa Jung, Jiyun Ahn

**Affiliations:** 1 Research Group of Natural Material and Metabolism, Korea Food Research Institute, Wanju, South Korea; 2 Department of Food Science and Technology, Jeonbuk National University, Jeonju-Si, South Korea; 3 Department of Food Biotechnology, University of Science and Technology, Daejeon, South Korea; 4 Department of Biotechnology, College of Life Sciences and Biotechnology, Korea University, Seoul, South Korea

**Keywords:** sarcopenia, obesity, microRNA, mitochondria, myogenesis

## Abstract

Post-menopausal conditions exacerbate the biological aging process and this is often accompanied by visceral adiposity with sarcopenia. Mitochondrial impairment is a hallmark of frailty and sarcopenia in the elderly. However, the exact mechanism underlying the development of obesogenic sarcopenia and the involvement of mitochondria remains unclear. This study confirmed that there is a decline in muscle mass and function as well as mitochondrial dysfunction in the quadriceps of ovariectomized (OVX) mice. To investigate the role of microRNA (miRNA) in this process, we performed miRNA and mRNA arrays and found that miR-141-3p directly targets and downregulates FK506 binding protein 5 (Fkbp5) and Fibin. Overexpression of miR-141-3p decreased mitochondrial function and inhibited myogenic differentiation in C2C12 cells. These effects were mediated by Fkbp5 and Fibin inhibition. Conversely, knockdown of miR-141-3p increased mitochondrial respiration and enhanced myogenesis. Treatment with β-estradiol effectively reversed the palmitic acid-induced upregulation of miR-141-3p and subsequent downregulation of Fkbp5 and Fibin. In conclusion, miR-141-3p is upregulated in OVX mice, and this is associated with mitochondrial dysfunction through inhibition of Fkbp5 and Fibin. These findings suggest that inhibiting miR-141-3p could be a therapeutic target for alleviating obesogenic sarcopenia.

## INTRODUCTION

Age-related changes in body composition are related to the incidence of obesity, osteoporosis, and sarcopenia [1]. Excess fat is a risk factor for loss of muscle mass and the concurrence of these conditions is defined as sarcopenic obesity [2]. The risk of sarcopenic obesity is higher in aging women; the prevalence of sarcopenic obesity is 48.0% in females over 80 years of age, versus 27.5% in males [3, 4]. In particular, after menopause, women undergo dramatic changes including an increase in body weight and fat mass and a decrease in lean body mass due to altered hormonal levels [4]. These changes can be attenuated by estrogen treatment, which modulates inflammation in skeletal muscle [5]. Ovariectomized (OVX) rodents are a good experimental model for sarcopenic obesity because they exhibit an obesity phenotype along with sarcopenia [6].

Mitochondria are important organelles that produce ATP through oxidative phosphorylation by the mitochondrial respiratory chain. Additionally, they serve a critical role in modulating energy supply, reactive oxygen species signaling, and intrinsic apoptosis pathways. Mitochondria from skeletal muscle of obese subjects are characterized by a small size, reduced content, impaired oxidative capacity, and increased ROS production [7]. For this reason, mitochondrial dysfunction is the most frequently implicated mechanism of aging muscle atrophy [8]. Previously, an examination of skeletal muscle mitochondrial function of OVX rats demonstrated that mitochondrial dysfunction is related to the development of obesity [9]. However, the exact mechanisms underlying OVX-induced obesity and mitochondrial dysfunction are unknown.

MicroRNAs (miRNAs) are small noncoding RNAs that regulate numerous gene networks and signaling pathways. MiRNAs bind to mRNA and regulate gene expression by degrading the mRNA or inhibiting protein translation [10]. MyomiRs refer to miRNAs that are specifically expressed in muscle and typically control myogenic differentiation and muscle tissue homeostasis [11]. Accumulating evidence has suggested that miRNAs play an important role in age-related changes in skeletal muscle [12, 13]. An analysis of skeletal muscle samples from young and old subjects identified miRNAs linked to muscle protein synthesis and regeneration [14]. During myogenic differentiation, miR-1 and miR-206 promote differentiation, whereas miR-133 inhibits differentiation [15, 16]. Nonmuscle-specific miRNAs such as miR-696, miR-29, miR-378, and miR-92b have also been associated with myogenesis through targeting PGC1α, histone deacetylase 4, bone morphogenic protein 2, and MEF2, respectively [17–20]. Mitochondria-related miRNAs, encoded by the mitochondrial or nuclear genome, have emerged as key regulators of cell metabolism and can modulate mitochondrial dynamics [21].

In the present study, we investigated the role of miRNAs in the etiology of sarcopenic obesity under menopause conditions. For this, we analyzed miRNA profiles and found differentially expressed miRNAs in skeletal muscle of OVX versus control mice. Using a functional study, we identified putative miRNA targets and validated miRNA-mRNA interactions. Moreover, we examined the possibility that miRNAs may mediate the effect of estrogen on skeletal muscle.

## RESULTS

### Sarcopenic obesity in OVX mice

**Figure 1:**
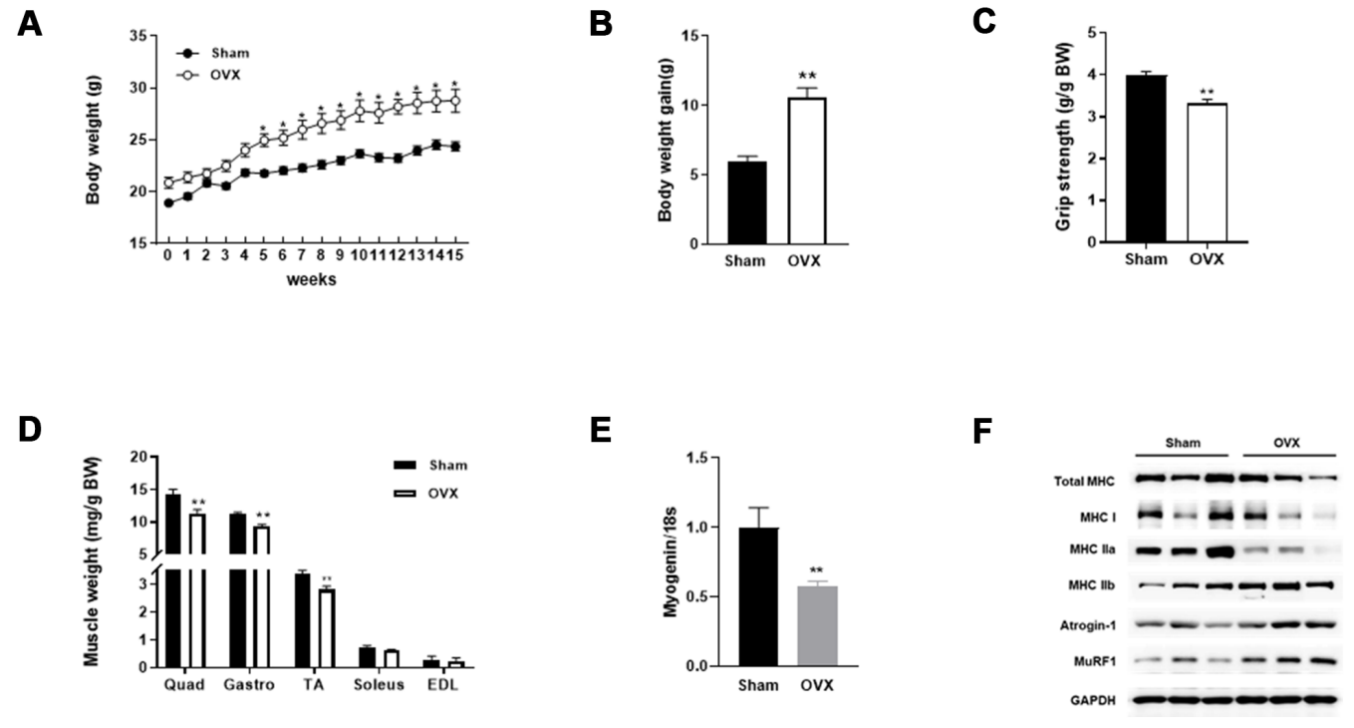
**OVX induces sarcopenic obesity in mice.** (**A**) Change of body weight during the experimental period. (**B**) Body weight gain of Sham and OVX mice after 15 weeks. (**C**) Average forelimb grip strength in Sham and OVX mice normalized by body weight. (**D**) Comparison of OVX muscle mass with Sham. (**E**) Relative mRNA expression of myogenin. Data are means ± SEM. *P<0.05, **P<0.01; unpaired two-tailed Student’s t-test. (**F**) Protein expression of genes related to muscle type transition (total MHC, MHC 1, MHC IIa, and MHC IIb) and muscle atrophy (Atrognin-1 and MuRF1).

Compared with sham-operated control mice (Sham), OVX mice showed increased weight gain and decreased grip strength and muscle weight 15 weeks post-surgery (Figure 1A–1D). Expression of myogenin mRNA was decreased in the quadriceps of OVX mice (Figure 1E). We measured myosin heavy chain protein levels and found a decrease in total and oxidative fiber (I and IIa) and an increase in glycolytic fiber (IIb) (Figure 1F). Interestingly, the OVX-induced decrease in MHC was more pronounced in slow-twitch fibers. Additionally, a significant increase was observed in atrogin-1 and MuRF1 protein expression in OVX mice.

The infiltration of ectopic fat in skeletal muscle is associated with reduced strength and performance of physical activity upon aging [22]. We compared the lipid content of skeletal muscle between Sham and OVX mice. As shown in Table 1, total lipid content, total cholesterol, and triglyceride levels were significantly increased in OVX mice. These data indicate that OVX evokes obesogenic sarcopenia in mice.

**Table 1:** Muscle lipid profile.

	**Sham**	**OVX**
Muscle weight (mg/BW)	14.31 ± 0.73	11.36 ± 0.53^*^
Total lipid (mg/g)	46.94 ± 4.73	71.69 ± 7.35^*^
TG (mg/g)	8.95 ± 1.58	31.62 ± 4.63^*^
TC (mg/g)	1.35 ± 0.05	2.05 ± 0.28^*^

Data are means ± SEM. *P<0.05, **P<0.01, ***P<0.001; two-tailed Student’s t-test.

### OVX evokes mitochondrial dysfunction

**Figure 2:**
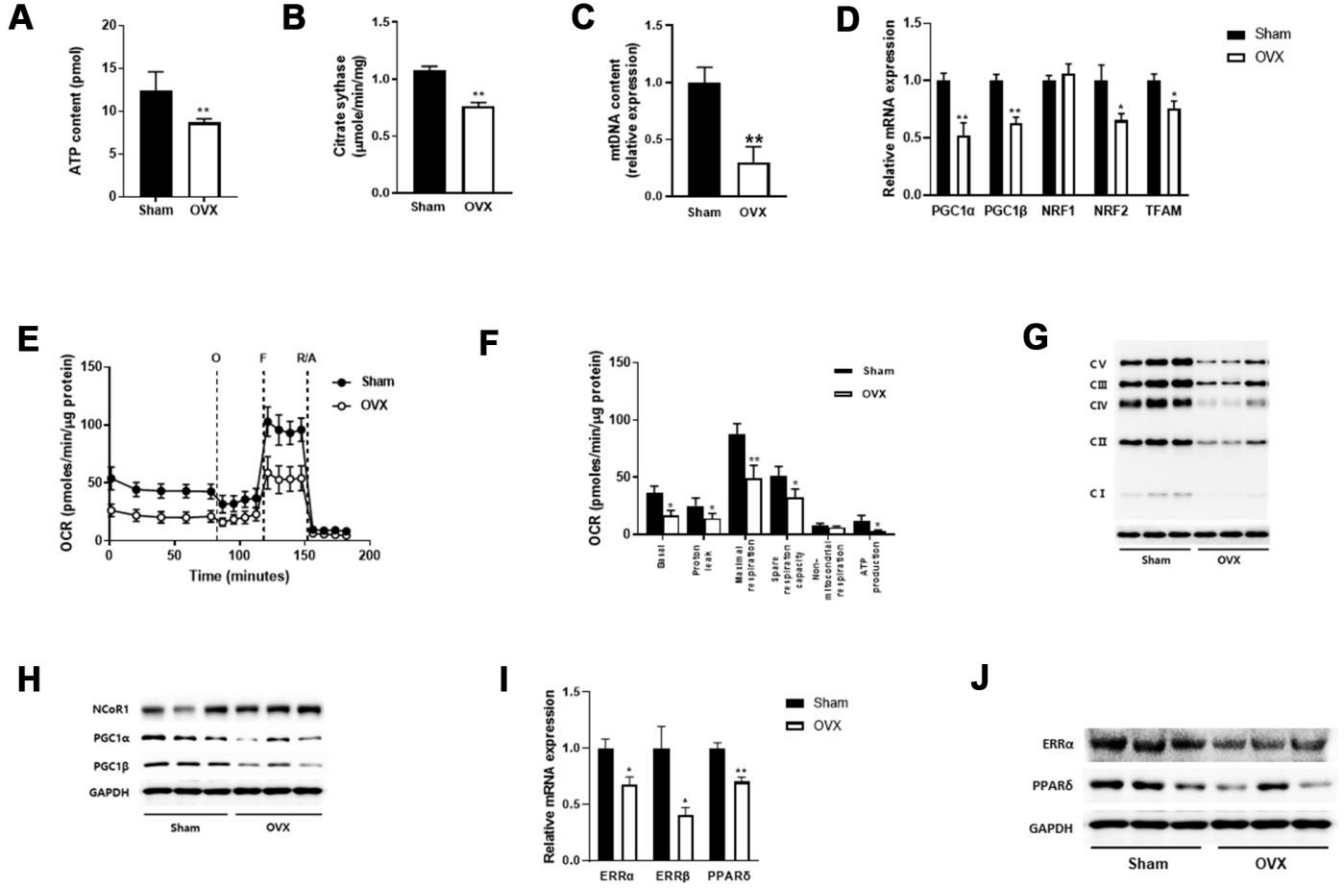
**OVX impairs mitochondrial function.** (**A**, **B**) ATP content (**A**) and citrate synthase activity (**B**) in quadriceps muscle (n=8 mice per group). (**C**) Mitochondrial DNA content evaluated by the ratio of a mitochondrial encoded gene (Cox5b) and a nuclear-encoded gene (18S). (**D**) Relative mRNA expression of genes involved in mitochondrial biogenesis. (**E**) Extracellular flux analysis in EDF fiber from Sham or OVX mice (n=6). OCR was measured before (basal) and after successive addition of oligomycin (O) to determine ATP-linked respiration, carbonyl cyanide *p*-(trifluoromethoxy) phenylhydrazone (FCCP, F) to examine the maximal respiration, rotenone along with antimycin (R/A) to assess non-mitochondrial respiration. (**F**) Average OCR values were compared between Sham and OVX mice. Data are means ± SEM. *P<0.05, **P<0.01; unpaired two-tailed Student’s t-test. (**G**) The levels of OXPHOS proteins in mitochondrial fraction. (**H**) Western blot analysis for NCoR1 and PGC1α/β. (**I**, **J**) The mRNA (**I**) and protein (**J**) expressions of ERR and PPARδ.

Alterations in skeletal muscle lipid metabolism are related to mitochondrial defects [23]. Thus, we measured the effect of OVX on mitochondrial function in the quadriceps muscle. We observed decreases in ATP content and the mitochondrial enzyme activity such as citrate synthase in OVX mice compared with those in control mice (Figure 2A, 2B). Mitochondrial contents were reduced in OVX mice (Figure 2C), which was associated with downregulation of peroxisome proliferator-activated receptor-γ coactivator 1α/β (PGC-1α/β) and its downstream targets nuclear respiratory factor 1 and 2 (NRF-1 and NRF-2), and mitochondrial transcription factor A (TFAM), all of which are involved in the regulation of mitochondrial biogenesis (Figure 2D).

We then performed extracellular flux analysis in isolated extensor digitorum longus (EDL)fibers using an XF24 analyzer. A significant reduction was noted in the overall oxidative phosphorylation capacity in OVX mice compared with that in control mice (Figure 2E). After sequential addition of oligomycin, FCCP, and rotenone in combination with antimycin A, mitochondrial respiration such as basal, proton leak, maximal respiration, spare respiratory capacity, and ATP production were significantly decreased in OVX mice (Figure 2F). Regarding mitochondrial complex protein expression, we observed a significant decrease in OXPHOS enzyme levels in OVX mice compared with those in control mice (Figure 2G). These results suggest that OVX evokes mitochondrial dysfunction in the skeletal muscle of mice.

Nuclear receptor corepressor 1 (NCoR1) and PGC1α regulate the transcriptional activity of nuclear receptors, peroxisome proliferator-activated receptor delta (PPARδ) and estrogen-related receptor α (ERRα), with opposing effects to regulate oxidative phosphorylation [24]. To examine the involvement of NCoR1 and PGC1α in reducing OCR in OVX, we performed western blot analysis and observed opposite expression between NCoR1 and PGC1α (Figure 2H). qRT-PCR and western blot data also showed that ERRα/β and PPARδ were downregulated in OVX mice (Figure 2I, 2J).

### miR-141-3p is upregulated in OVX and directly targets FK506-binding protein 5 (Fkbp5) and Fibin

**Figure 3:**
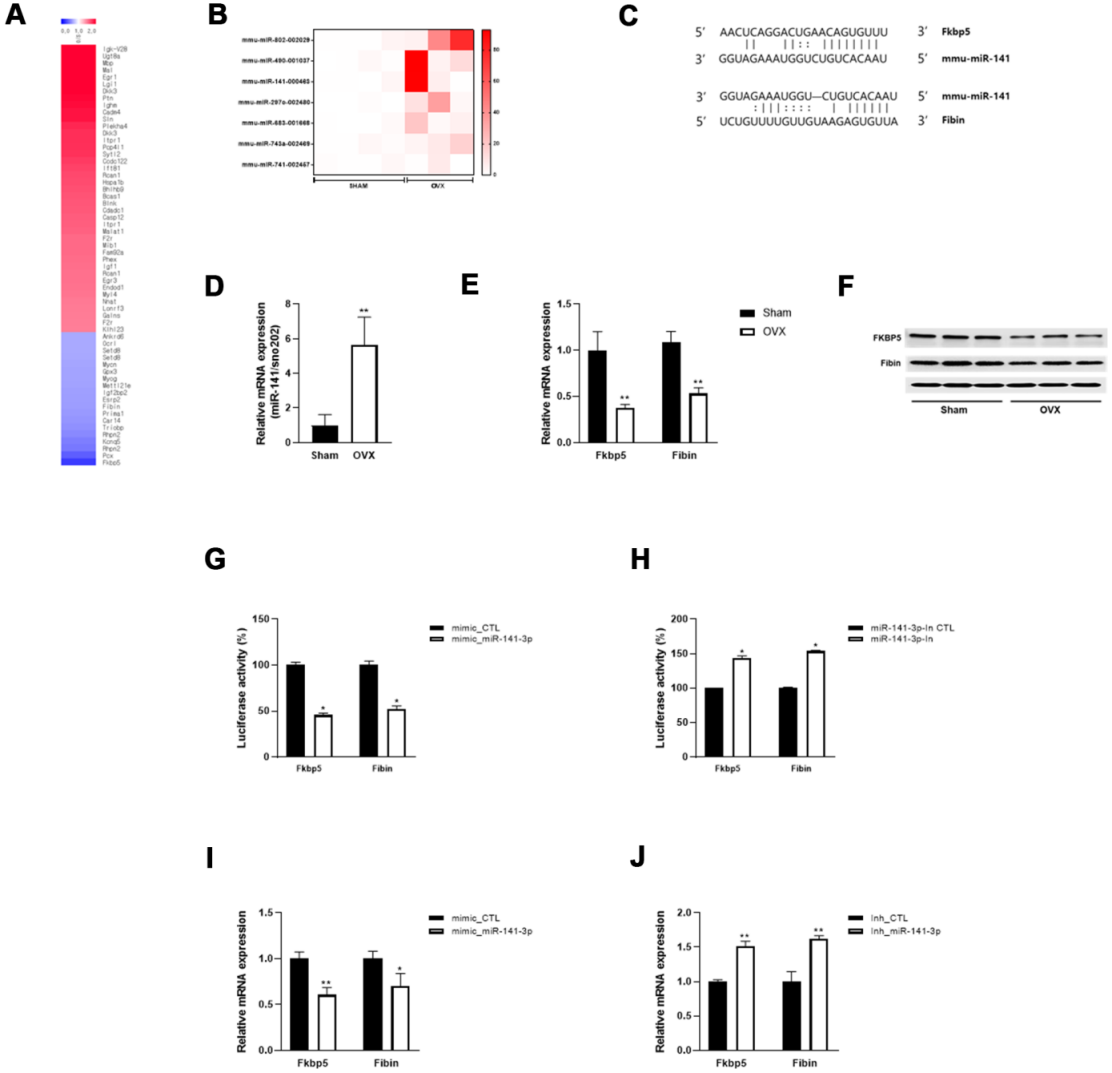
**miR-141-3p targets Fkbp5 and Fibin.** (**A**) Heatmap of significantly up and down-regulated differentially expressed mRNA in OVX mice. (**B**) Differentially expressed miRNA in OVX mice. (**C**) The putative binding site between miR-141-3p and its targets, Fkbp5 and Fibin. (**D**) The upregulation of miR-141-3p in OVX mice was validated by qRT-PCR. (**E**, **F**) The expressions of Fkbp5 and Fibin in mRNA (**E**) and protein (**F**) levels. (**G**, **H**) A reporter vector containing Fkbp5 or Fibin 3’UTR was transfected along with miR-141-3p mimic (**G**) or inhibitor (**H**). The luciferase activity was normalized to renilla luciferase activity. (**I**, **J**) Measurement of Fkbp5 or Fibin expressions after exposure miR-141-3p mimic (**I**) or inhibitor (**J**). Data are means ± SEM. *P<0.05, **P<0.01; unpaired two-tailed Student’s t-test.

To investigate the regulatory mechanisms that underlie OVX-induced muscle atrophy and mitochondrial dysfunction, we performed microarray analysis and identified mRNAs that were differentially expressed between Sham and OVX mice (Figure 3A). Fkbp5 showed the most significant decrease in expression. Subsequently, the miRNA array data revealed seven miRNAs that were differentially expressed between the two groups (log2 fold change > 10) (Figure 3B). To predict miRNA target genes, we further compared the differentially expressed mRNAs and miRNAs and identified Fkbp5 and Fibin to have a binding sequence for miR-141-3p (Figure 3C).

To validate the expression of miR-141-3p and its targets, we performed qRT-PCR and observed an upregulation of miR-141-3p and downregulation of Fkbp5 and Fibin in OVX mice (Figure 3D, 3E). Western blot analysis confirmed the decrease in Fkbp5 and Fibin at the protein level (Figure 3F). We performed a luciferase binding assay to determine whether miR-141-3p binds to these predicted targets. Exposure to a miR-141-3p mimic significantly reduced the luciferase activity of the reporter containing the 3´UTR of Fkbp5 and Fibin (Figure 3G). Conversely, a miR-141-3p inhibitor significantly increased luciferase activity (Figure 3H). To further investigate whether miR-141-3p regulates Fkbp5 and Fibin, we transfected C2C12 cells with the miR-141-3p mimic and found that Fkbp5 and Fibin expression was decreased at the mRNA level. Conversely, treatment with the miR-141-3p inhibitor increased Fkbp5 and Fibin expression (Figure 3J). These results suggest that miR-141-3p may directly bind to Fkbp5 and Fibin and negatively regulate their expression.

### miR-141-3p inhibits mitochondrial function and myogenic differentiation

**Figure 4:**
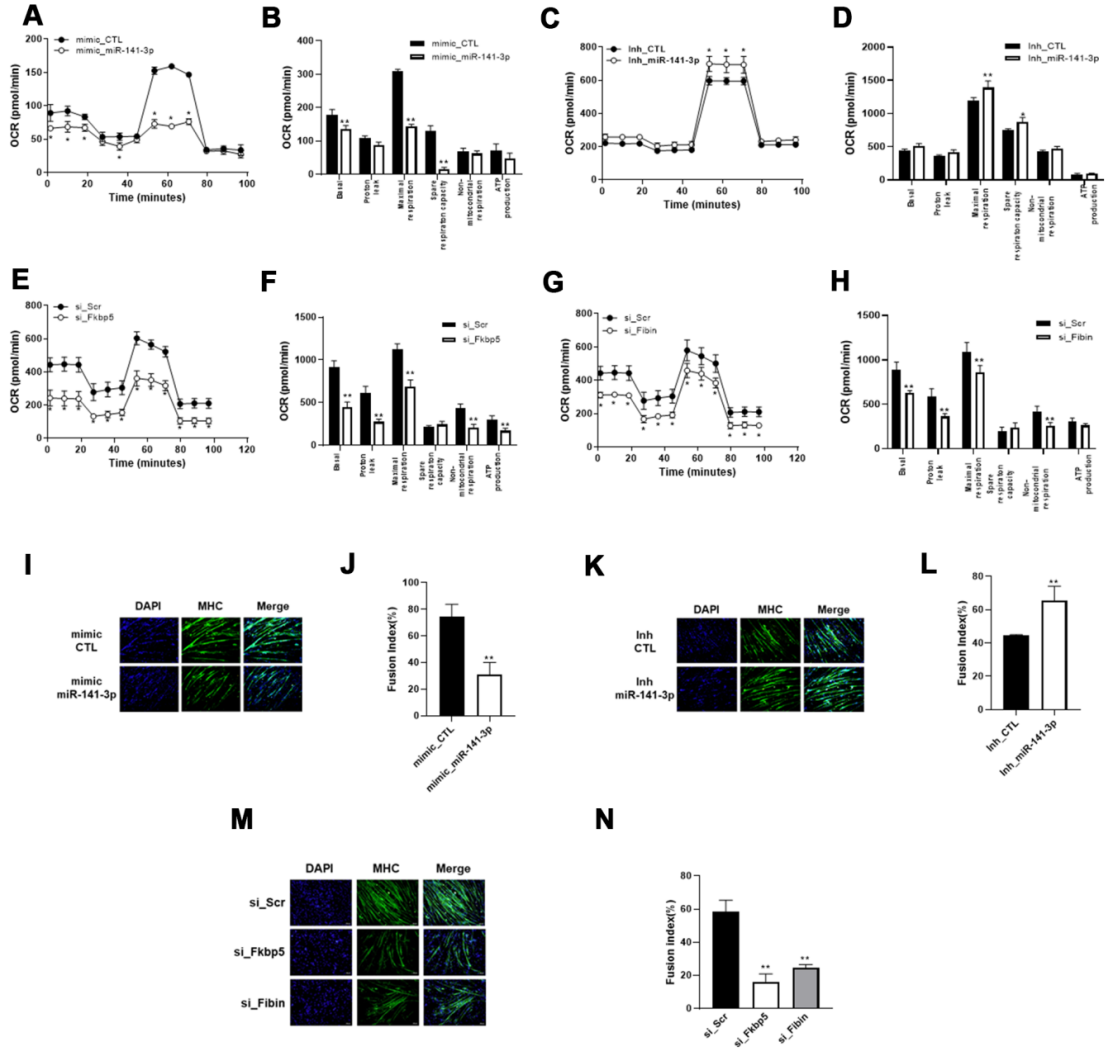
**miR-141-3p induces mitochondrial dysfunction and impairs muscle differentiation through downregulation of Fkbp5/Fibin.** (**A**–**D**) Mitochondrial respiration was measured in miR-141-3p overexpressed (**A**) or silenced (**C**) C2C12 cells. Average OCR values were indicated in miR-141-3p overexpressed (**B**) or silenced (**D**) C2C12 cells. (**E**, **F**) Mitochondrial respiration (**E**) and average OCR value (**F**) were measured in Fkbp5 knockdown C2C12 cells. (**G**, **H**) Mitochondrial respiration (**G**) and average OCR value (**H**) were measured in Fibin knockdown C2C12 cells. (**I**–**L**) C2C12 cells were treated with miR-141-3p mimic or miR-141-3p inhibitor and induced to differentiate. The expression of MHC was analyzed by immunostaining (**I**–**K**). Cells were stained with anti-MHC (green) and DAPI (blue) for nuclei detection. Fusion index of miR-141-3p mimic (**J**) or miR-141-3p inhibitor (**L**) was calculated. Data are means ± SEM. *P<0.05, **P<0.01; unpaired two-tailed Student’s t-test. (**M**) Fkbp5 or Fibin silenced C2C12 cells were differentiated and immunostaining was conducted to measure the expression of MHC. (**N**) Fusion index of Fkbp5 or Fibin silenced C2C12 cells. Data are means ± SD.**P<0.01 vs si_Scr; one-way ANOVA with post hoc Dunnett’s multiple comparison test.

We hypothesized the miR-141-3p may play a role in the reduced mitochondrial function and muscle atrophy by regulating Fkbp5 and Fibin. To test this, we established miR-141-3p-overexpressing cells and examined the effects on mitochondrial respiration compared with control cells. As shown in Figure 4A, miR-141-3p-overexpressing C2C12 cells exhibited a significantly lower OCR than control cells. Basal and maximal respiration were significantly inhibited by miR-141-3p overexpression. In contrast, miR-141-3p knockdown caused an increase in OCR and maximal respiration (Figure 4C, 4D). To better understand the role of miR-141-3p in mitochondrial metabolism, we performed siRNA for the miR-141-3p targets, Fkbp5, and Fibin, and measured OCR (Figure 4E–4H). As observed in miR-141-3p-overexpressing cells, silencing of Fkbp5 and Fibin caused a reduction in OCR and inhibited basal and maximal respiration. These results imply that miR-141-3p upregulation reduces the mitochondrial respiration capacity of OVX muscle via downregulation of Fkbp5 and Fibin.

Next, we induced myogenic differentiation in miR-141-3p-overexpressing C2C12 cells to assess the functional significance of miR-141-3p in myogenesis (Figure 4I). Immunostaining for MHC showed that overexpression of miR-141-3- inhibited myoblast differentiation. We found that transfection of the miR-141-3p mimic decreased the fusion index to 41.7% compared with control cells (Figure 4J). In contrast, Mir-141-3p knockdown led to an increase in myoblast differentiation and an increase in the fusion index compared with control cells (Figure 4K, 4L). To confirm the anti-myogenic effect of miR-141-3p, we knocked down expression of the miR-141-3p targets, Fkbp5 and Fibin, in C2C12 cells and then induced their differentiation (Figure 4M, 4N). Silencing of Fkbp5 and Fibin led to a marked inhibition in myogenic differentiation and a reduced fusion index. Together, these results demonstrate that miR-141-3p inhibits myoblast differentiation by inhibiting Fkbp5 and Fibin.

### β-estradiol (E2) restores the PA-evoked decrease of Fkbp5 and Fibin

Previously, it was reported that OVX decreases estrogen receptor 1 (ESR1) expression and increases perilipin2 (PLIN2) and fatty acid synthase (FASN) expression at both the mRNA and protein level in skeletal muscle of rats [25].

**Figure 5:**
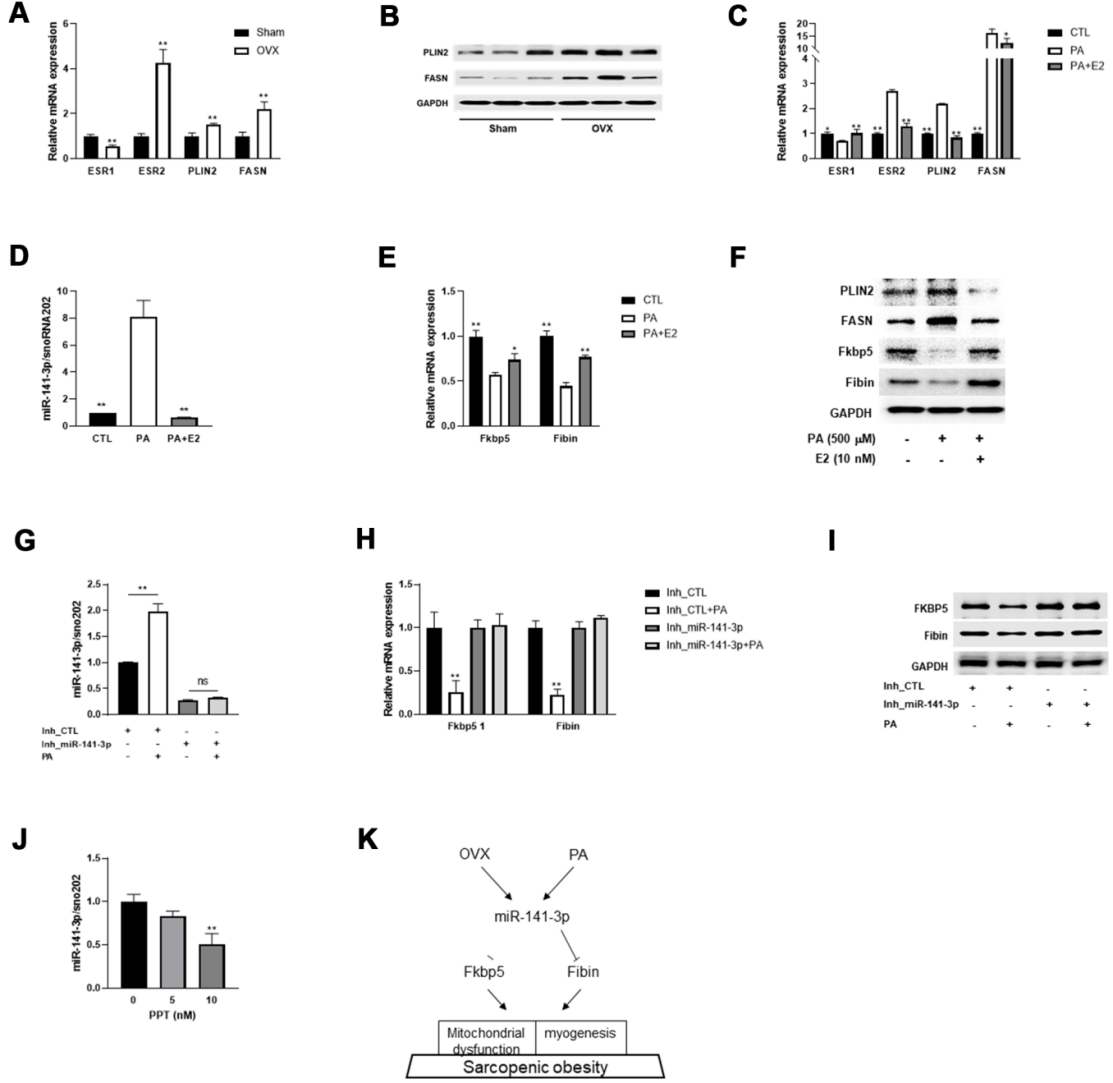
**E2 reverses miR-141-3p/Fkbp5 and Fibin signaling.** (**A**) Relative mRNA expressions of estrogen receptor 1 or 2 (ESR1/2) and lipid metabolism associated genes (PLIN2 and FASN). Data are means ± SEM. *P<0.05, **P<0.01; unpaired two-tailed Student’s t-test. (**B**) Western blot analysis for PLIN2 and FASN. (**C**) Relative mRNA expression of OVX-related genes in PA or PA+E2 treated C2C12 myotubes. (**D**) The miR-141-3p expression in PA or PA+E2 treated C2C12 myotubes. (**E**, **F**) Expressions of Fkbp5 and Fibin in mRNA (**E**) and protein (**F**) levels from PA or PA+E2 treated C2C12 myotubes. *P<0.05, **P<0.01 vs PA-treated C2C12 myotubes. (**G**–**I**) The effect of miR-141-3p knockdown on PA induced miR-141-3p expression (**G**), Fkbp5 and Fibin expressions in mRNA and protein levels (**H**, **I**, respectively). **P<0.01 vs Inh_CTL-treated C2C12 cells. (**J**) After incubation for 24h with propylpyrazoletriol (PPT), an ESR1 agonist, the expression of miR-141-3p was measured in C2C12 cells. **P<0.01 vs control. Data are means ± SD. one-way ANOVA with post hoc Dunnett’s multiple comparison test. (**K**) The miR-141-3p/Fkbp5 and Fibin signaling in sarcopenic obesity.

To mimic lipid overload to skeletal muscle, we incubated C2C12 myotubes with palmitic acid (PA) and observed similar gene expression patterns as were seen in differentiated C2C12 cells. Consistent with this, we also observed downregulation of ESR1 and upregulation of PLIN2 and FANS in the quadriceps of OVX mice (Figure 5A, 5B). Treatment with E2 significantly ameliorated PA-evoked changes in gene expression, including ESR1/2, PLIN2, and FANS (Figure 5C).

Subsequently, we measured the expression of miR-141-3p and its two targets and found that exposure to PA induced the upregulation of miR-141-3p and subsequent downregulation of Fkbp5 and Fibin (Figure 5D–5F). To investigate whether treatment with E2 ameliorated the OVX-evoked induction of miR-141-3p, we co-treated C2C12 myotubes with PA and E2 and measured miR-141-3p expression. Interestingly, E2 treatment effectively reversed the PA-evoked dysregulation of miR-141-3p. In addition, the decrease in Fkbp5 and Fibin by PA was recovered by E2 treatment at both the mRNA and protein level. To clarify whether the PA-evoked decrease in Fkbp5 and Fibin expression was mediated by miR-141-3p, we co-treated miR-141-3p inhibitor-treated C2C12 cells with PA and measured Fkbp5 and Fibin expression. Results showed that the PA-evoked miR-141-3p upregulation was abolished in miR-141-3p-silenced C2C12 cells (Figure 5G). The loss of miR-141-3p induction did not affect Fkbp5 and Fibin levels at either the mRNA or protein level (Figure 5H, 5I). Treatment with PPT, an ESR1 agonist, caused a dose-dependent decrease in miR-141-3p expression (Figure 5J). These data suggest that miR-141-3p may play a role in the protective effects of E2 on OVX.

## DISCUSSION

OVX is a good model for investigating the influence of estradiol deficiency on energy metabolism [26]. OVX animals exhibit an increase in body weight and a decrease in body mass and energy expenditure, and thus can be used as a preclinical model for sarcopenic obesity, even though it does not exactly recapitulate aging-associated sarcopenia [27]. The present study demonstrated an important role for miR-141-3p in the development of obesogenic sarcopenia in OVX mice. OVX induced an upregulation of miR-141-3p and subsequent downregulation of Fkbp5 and Fibin. This cascade is associated with impaired mitochondrial function and decreased myogenesis in OVX animals (Figure 5K).

The development of obesity and insulin resistance is related to skeletal muscle mitochondrial dysfunction in OVX rats [9]. However, they displayed decreased complex I+II mediated respiration in the soleus muscle but not in the gastrocnemius muscle 8 weeks after OVX surgery. Furthermore, when normalized to citrate synthase activity, a reduced O_2_ consumption in muscles could not be detected. Unlike the previous study, we analyzed mitochondrial function in isolated EDL myotubes 15 weeks after OVX in mice. We observed a distinct reduction in OCR in the OVX group compared with the Sham group. Mitochondrial dysfunction was validated by reduced ATP content, reduced mitochondrial DNA content, and a decrease in the expression of mitochondrial respiratory complexes at the protein level. Mitochondrial biogenesis is mainly regulated by peroxisome proliferator-activated receptor-γ coactivator 1α (PGC1α), a transcriptional regulatory factor. PGC1α does not interact directly with DNA, but rather with transcription factors such as nuclear respiratory factor 1 and 2 (NRF-1 and NRF-2) [28]. This transcriptional activation leads to an increase in mitochondrial transcription factor A (TFAM), which is essential for mtDNA replication, transcription, and maintenance [29]. We found that mitochondrial biogenesis was inhibited in OVX via downregulation of the PGC1 family. NCoR1 and PGC1α compete for the transcriptional regulation of ERRα to regulate oxidative metabolism in skeletal muscle [24]. Our results show that NCoR1 was increased whereas PGC1α was decreased in the quadriceps muscle of OVX animals. These changes result in downregulation of ERRα/β and PPARδ, which consequently reduces mitochondrial function.

Previously, miR-141-3p, a mitochondria-related miRNA [21], was reported to suppress colorectal cancer by targeting tumor necrosis factor receptor-associated factor 5 [30]. MiR-141-3p is upregulated in hepatic steatosis and induces oxidative stress by targeting PTEN [31]. However, the role of miR-141-3p in the development of sarcopenic obesity remains unclear. In hepatocellular carcinoma, ESR1 suppresses circular RNA-SMG1.72 and this leads to the downregulation of miR-141-3p, eventually inhibiting cell invasion [32]. This observation raised the possibility that miR-141-3p, one of the downstream molecules of ESR1, could be regulated by ESR1 condition. Herein, we observed an upregulation of miR-141-3p in ESR1-reduced OVX muscle, whereas an increase in ESR1 by E2 exposure induced a downregulation of miR-141-3p. Also, treatment with an ESR1 agonist suppressed miR-141-3p expression. We therefore conclude the ESR1 directly downregulates miR-141-3p in OVX muscle.

Fkbp5 is an important modulator of stress responses, acting as a co-chaperone to modulate glucocorticoid-receptor activity [33]. Although Fkbp5 is most commonly discussed in the context of regulating the stress response, it also interacts with ERS1 to promote ESR activity [34]. An increase in Fkbp5 was observed in the skeletal muscles of hypergravity-induced muscle hypertrophy mice [35]. Moreover, Fkbp5 overexpression increased Akt and p70 S6 kinase phosphorylation, which are involved in the muscle protein synthesis pathway, and decreased atrogin-1 and MuRF1 expression, which regulate muscle protein degradation. We observed a decrease in Fkbp5 in muscle tissues of sarcopenic obesity mice. It is possible that a decrease in Fkbp5 could exacerbate muscle loss through the inhibition of protein synthesis and an increase in muscle atrophy.

Fibin was initially discovered in zebrafish as an essential secreted signaling molecule, and is reported to be a novel growth factor in zebrafish and mouse embryos [36]. A qRT-PCR analysis of the tissue distribution of Fibin found that expression is high in the cerebellum and skeletal muscle [37]. However, the role of Fibin in myogenesis and mitochondrial function had not yet been reported. Our results represent the first demonstration of a crucial role for Fibin in myogenesis and mitochondrial function.

Mitochondria play a potential regulatory role in myogenesis and mitochondrial dysfunction, including morphological alterations, energy stress via reduced ATP, and enhanced ROS production, all of which contribute to the development of muscle atrophy [38]. Knockdown of Fkbp5 or Fibin impaired mitochondrial oxidative phosphorylation and inhibited myogenic differentiation, suggesting that they are important regulators of myogenic differentiation via their control of mitochondrial function.

In conclusion, our findings identify a novel signaling pathway that may regulate muscle atrophy in OVX via miRNA. MiR-141-3p is upregulated in OVX muscle, and it negatively regulates Fkbp5 and Fibin via direct binding to their 3´UTR. Mitochondrial function is also impaired by miR-141-3p, and myogenesis is inhibited via downregulation of Fkbp5 and Fibin. Thus, the use of either miR-141-3p inhibitors or Fkbp5/Fibin agonists could be an effective therapeutic strategy for ameliorating sarcopenic obesity.

## MATERIALS AND METHODS

### Ovariectomy-induced skeletal muscle atrophy

Eight week-old female C57/BL6 mice were sham-operated or bilaterally ovariectomized and left for 15 weeks to develop obesogenic sarcopenia. Skeletal muscle tissues were harvested and used for subsequent experiments. All animal experiments were performed according to procedures approved by the Institutional Animal Care and Use Committee of the Korea Food Research Institute (KFRI-M-18021).

### Grip strength analysis

Forelimb grip strength of mice was measured using a grip strength meter (Bioseb, Chaville, France). Mice grasped the grid with their forelimb and the mean of five measurements for each animal was calculated. Skeletal muscle strength is expressed in grams.

### Quantitative RT-PCR

An RNeasy mini kit and NucleoSpin RNA plus (Macherey-Nagel, Germany) were used to isolate total RNA from tissues and C2C12 cells, respectively. cDNA synthesis was performed using a Bio-Rad thermocycler (CA, USA) and a ReverTra Ace qPCR RT Master Mix (Toyobo, Japan). To measure mRNA expression, SYBR green quantitative PCR amplifications was used to perform qRT-PCR using a Viia 7 system (Applied Biosystems, CA, USA). Relative mRNA expression levels were normalized to 18S and GAPDH. miRNA expression was determined using a TaqMan MicroRNA Assay (Thermo Fisher Scientific, MA, USA) following the manufacturer’s protocol. cDNA was reverse transcribed using a TaqMan MicroRNA Reverse Transcription kit (Thermo Fisher Scientific). A TaqMan Universal Master MIX II, no UNG (Thermo Fisher Scientific), and miRNA primers were amplified using a ViiA 7 system. Relative miRNA expression levels were normalized to sno202 using the 2^−ΔΔCt^ method. Primer sequences are listed in Table 2.

**Table 2:** Primer sequence used for qRT-PCR.

	**Primer (5ʹ→3ʹ)**	
**Gene**	**Forward**	**Reverse**
MyoG	GAGACATCCCCCTATTTCTACCA	GCTCAGTCCGCTCATCGCC
PGC1alpha	CACCAAACCCACAGAAAACAG	GGGTCAGAGGAAGAGATAAAGTTG
PGC1beta	TCCTGTAAAAGCCCGGAGTAT	GCTCTGGTAGGGGCAGTGA
NRF1	AATGTCCGCAGTGATGTCC	GCCTGAGTTTGTGTTTGCTG
NRF2	TGAAGTTCGCATTTTGATGGC	CTTTGGTCCTGGCATCTCTAC
TFAM	CACCCAGATGCAAAACTTTCAG	CTGCTCTTTATACTTGCTCACAG
ERRalpha	CCACCCCCTGTTTTGCAT	CTGGCTGCTTGTAGGACACA
ERRbeta	ACGGCTGGATTCGGAGAAC	TCCTGCTCAACCCCTAGTAGATTC
PPARbeta	TAGAAGCCATCCAGGACACC	CCGTCTTCTTTAGCCACTGC
Fkbp5	TGGTGTTCGTTGTTGGGGAA	AACTTAGGCTTCCCGGCTTC
Fibin	CCGGGGGAACCTTTTTCCTT	CTGGCATTTCTCGGGGTCAT
ESR1	TCCTAACTTGCTCCTGGACAGG	GTAGCCAGCAACATGTCA
ESR2	TTCTTTCTCATGTCAGGCACA	CTCGAAGCGTGTGAGCATT
PLIN2	CCCGCAACCTGACCCAGCAG	CGCCTGCCATCACCCCCAAG
FASN	GGAGGTGGTGATAGCCGGTAT	TGGGTAATCCATAGAGCCCAG
Cox2	ATAACCGAGTCGTTCTGCCA	GCTTGATTTAGTCGGCCTGG
18S	CTCAACACGGGAAACCTCAC	CGCTCCACCAACTAAGAACG
GAPDH	CATCTTCCAGGAGCGAGACC	TGAAGTCGCAGGAGACAACC

### Western blotting

Total protein was extracted from C2C12 cells and skeletal muscle tissue using RIPA buffer (Thermo Fisher Scientific) and the mitochondrial fraction was prepared using a mitochondria isolation kit for tissue (Thermo Fisher Scientific) following the manufacturer’s protocol. Protein concentration was measured using a Pierce BCA Protein Assay Kit (Thermo Fisher Scientific). Protein samples were loaded and separated on a 12% SDS PAGE gel and transferred to a polyvinylidene difluoride (PVDF) membrane (Bio-Rad). Non-specific sites were blocked with 5% skim milk in TBST and the membrane was incubated with primary antibodies overnight at 4° C, followed by incubation with HRP-conjugated secondary antibodies for 1 h at room temperature. Antibodies specific to atrogin-1, MuRF1, NCoR1, PGC1α/β, Fkbp5, and PLIN2, as well as a total OXPHOS antibody cocktail were obtained from Abcam (Cambridge, UK). Total and MHC subtype antibodies were supplied by DSHB (IA, USA). Anti-PLIN2 and anti-GAPDH were purchased from Cell Signaling Technologies (MA, USA).

### Measurement of muscle lipid

Total lipid content in the gastrocnemius muscle was estimated after extraction using the Folch method [39]. Briefly, the gastrocnemius was homogenized with 0.9% NaCl and kept overnight at 4° C in Folch solution. Tissues were centrifuged at 750g at 4° C for 15 min. The supernatant was filtered and evaporated with nitrogen. The final lipid fraction was dissolved in chloroform and used for the quantification of neutral lipid, triacylglycerol, and total cholesterol. Total cholesterol and triglyceride were measured using the total cholesterol test and triglyceride test (MBL, Korea) following the manufacturer’s protocol.

### Measurement of mitochondrial function

ATP contents were detected using an ATP synthase enzyme activity microplate assay kit (Abcam) following the manufacturer’s protocol. ATP content was determined using a luciferin-luciferase based bioluminescence assay, and was expressed as concentration in pmol. Citrate synthase activity was determined using a Citrate Synthase Assay Kit (Sigma Aldrich, CA, USA) following the manufacturer’s protocol. Citrate synthase was expressed in μmol/min/mg total protein.

### Mitochondrial DNA content

Mitochondrial DNA was extracted from gastrocnemius muscle using an RNeasy mini kit (Qiagen, MD, USA) following the manufacturer’s protocol. Mitochondrial DNA was quantified using qRT-PCR by measuring Cox2 mRNA expression levels and normalizing to 18S.

### Oxygen consumption rate (OCR) measurement

EDL muscle was isolated and OCR was measured according to a previous study, with minor modifications [40]. Collagenase-digested single muscle fiber was seeded onto XF24 wells and OCR was measured using a Seahorse XF24 Analyzer (Agilent, CA, USA). This was followed by injections of the mitochondrial inhibitor oligomycin (1 μM), the mitochondrial uncoupler carbonyl cyanide 4-(trifluoromethoxy) phenylhydrazone (FCCP; 400 nM), pyruvate (10 mM), and finally rotenone to inhibit the mitochondrial complex completely. A BCA assay was performed to determine muscle protein and results were normalized to protein content. For C2C12 cells, after measuring basal respiration, oligomycin (1 μM), FCCP (4 μM), and rotenone (1 μM) were injected consecutively. Results were analyzed using Wave Controller Software (Agilent, CA, USA).

### Microarray profiling of miRNA and mRNA

For the mRNA array, total RNA was isolated from gastrocnemius muscle using an RNeasy mini kit, and quality and quantity were assessed using a Bioanalyzer 2100 (Agilent, CA, USA). Biotin-labeled cRNA was hybridized onto GeneChip® Mouse Genome 4 30 2.0 Arrays (Affymetrix, CA, USA). GeneChips were scanned using a Affymetrix GeneChip Scanner 3000 7G. Data were analyzed using a Robust Multi-Array Analysis. Normalized, log-transformed intensity values were then analyzed using GeneSpring GX 1 3. 1 Agilent Technologies. For the miRNA array, reverse transcription and qPCR were performed using ReverTra Ace qPCR RT Master Mix and MegaPlex primers according to the manufacturer’s protocol. The reverse transcription product (2.5 μL) was added to 22.5 μL preamplification mix containing MegaPlex Rodent Pool A PreAmp primers (Thermo Fisher Scientific). Preamplification products were added to a TaqMan Universal PCR Master Mix No UNG (Thermo Fisher Scientific) and loaded onto the TaqMan™ Rodent MicroRNA A Array (Thermo Fisher Scientific). Normalized expression levels were expressed as fold change between sham and OVX groups.

### Luciferase reporter assay

Plasmids containing the 3´UTR response element of Fkbp5 or Fibin were generated using a pMIR-REPORT miRNA expression reporter vector system (Thermo Fisher Scientific). Plasmid DNA and miR-141-3p mimic or inhibitor or CTL were transfected using Lipofectamine 2000 (Thermo Fisher Scientific). After 48 hours, luciferase activity was measured using a Secrete-Pair Dual Luminescence Assay kit (GeneCopodia, MD, USA) and was normalized to alkaline phosphatase activity.

### Cell culture

C2C12 cells were purchased from American Type Culture Collection (Manassas, VA, USA) and cultured in Dulbecco’s modified Eagle’s medium (DMEM) containing 10% fetal bovine serum (FBS), 100 U /mL penicillin and 100 μg/mL streptomycin (Invitrogen, Carlsbad, CA, USA) in a 5% CO_2_ incubator at 37° C. When cells reached 100% confluency, medium was changed to differentiation medium (DM, DMEM with 2% horse serum, 100 U/mL penicillin and 100 μg/mL streptomycin) every 2 days and cells were left to fully differentiate for 4 days. To induce atrophy in the differentiated C2C12 cells, the medium was replaced by medium containing 0.5 mM bovine serum albumin-conjugated sodium palmitate (PA, Sigma Aldrich) for 24 hours. E2 (10 nM, Sigma Aldrich) and PPT (Sigma Aldrich) were co-administered with PA.

### miR-141-3p mimic and inhibitor transfection

For the functional study, we examined the effects of miR-141-3p gain or loss of function on mitochondrial respiration and myogenic differentiation in C2C12 cells. To induce miR-141-3p overexpression, a double-stranded RNA oligonucleotide, miRIDIAN miR-141-3p mimic (Thermo Fisher Scientific), was selected. For miR-141-3p knockdown, the miRIDIAN hairpin inhibitor (Thermo Fisher Scientific), which has a novel structure that inhibits the function of endogenous miRNA, was used. The negative control oligonucleotide sequence was based on *Caenorhabditis elegans* miR-67. Oligonucleotides (1 pmol) were transfected using the Lipofectamine™ RNAiMAX system (Thermo Fisher Scientific), and miR-141-3p overexpression and inhibition were verified using qRT-PCR as described above.

### siRNA transfection

ON-TARGET plus SMART pool siRNAs for Fkbp5 and Fibin were purchased from Dharmacon (CO, USA) along with control siRNA. C2C12 cells were transfected with 1 pmol of Fkbp5 or Fibin siRNA using Lipofectamine 2000 (Thermo Fisher Scientific). After 24 h, cells were differentiated with DM.

### Immunofluorescence (IF) staining and quantification of fusion index

To confirm myogenic differentiation of C2C12 cells, we examined MHC expression via IF staining. Differentiated C2C12 cells were fixed with 4% paraformaldehyde in PBS and saponificated with 0.05% saponin in PBS at room temperature. The cells were blocked with 1% BSA in PBS for 30 min at room temperature then incubated overnight at 4° C with the primary antibody, MHC (DSHB, IA, USA). After washing, cells were incubated with an HRP-conjugated anti-mouse secondary antibody (Cell Signaling, MA, USA) for 1 h at room temperature. Cells were counterstained with DAPI for 1 min at room temperature. Images were taken with a microscope Olympus (Tokyo, Japan). Fusion index was calculated as the percentage of nuclei in MHC-stained cells compared with the total number of nuclei.

### Statistical analysis

Data were analyzed using GraphPad Prism version 8.0 (GraphPad Software, Inc., La Jolla, CA) and expressed as mean ± SD (*in vitro*) or SEM (*in vivo*). One-way ANOVA was used for statistical analysis followed by Dunnett’s multiple comparison test. A probability value of p < 0.05 was used as the criterion for statistical significance.
